# Improving access to free school meals for children in poverty in Northern Ireland

**DOI:** 10.23889/ijpds.v8i2.2345

**Published:** 2023-09-14

**Authors:** Nicole Gleghorne

**Affiliations:** 1 Queen's University, Belfast, Belfast, United Kingdom

## Objectives

Two thirds of children in poverty did not receive free school meals (FSM) between 2015 and 2019 in Northern Ireland (Department for Communities in Northern Ireland, 2022). This project aims to explore this discrepancy between eligibility and access to FSM and improve access to FSM for all children in poverty.

## Methods

Data from the Family Resources Survey (FRS) between 2017 and 2020 was obtained from the UK Data Service and FSM eligibility was calculated using the current criteria provided by the Department for Education in Northern Ireland. Cross-tabulations were used to explore the number of children in receipt of FSM compared to those eligible but not in receipt of FSM. Multilevel logistic regression models used FSM eligibility as the dependent variable and relative and absolute poverty before and after housing costs and number of siblings in a family as the independent variables, controlling for age and gender of the child.

## Results

Preliminary analysis suggests that increasing the income threshold for families with more than one child provides greater access to FSM for children in poverty. There is also evidence that using the OECD modified equivalisation to adjust household income for larger families improves the rate of FSM eligibility for children in relative and absolute poverty before and after housing costs. The findings aim to provide evidence on the most appropriate threshold and equivalisation calculation required to ensure all children in poverty are eligible for FSM, giving greater access to FSM and educational opportunities to those children who need it most.

## Conclusion

The project will inform the current Anti-Poverty Strategy which is under review in Northern Ireland highlighting the need to consider family size through equivalisation into the FSM eligibility criteria. It also emphasizes the importance of administrative data to inform policy on FSM and other educational opportunities in Northern Ireland.

